# Icariin attenuates the tumor growth by targeting miR-1-3p/TNKS2/Wnt/β-catenin signaling axis in ovarian cancer

**DOI:** 10.3389/fonc.2022.940926

**Published:** 2022-09-14

**Authors:** Yanjin Fu, Haiquan Liu, Mengsha Long, Linliang Song, Zuyu Meng, Shaozi Lin, Yiyao Zhang, JiaJia Qin

**Affiliations:** ^1^ School of Traditional Chinese Medicine, Jinan University, Guangzhou, China; ^2^ Huizhou Traditional Chinese Medicine Hospital, Guangzhou University of Traditional Chinese Medicine, Huizhou, Guangdong, China

**Keywords:** icariin, ovarian cancer, Wnt/β-catenin signaling, miR-1-3p, TNKS2

## Abstract

**Purpose:**

Despite various therapy advances, ovarian cancer remains an incurable disease for which survival rates have only modestly improved. Natural products are important sources of anti-cancer lead compounds. Icariin exhibited broad anti-cancer efficacy. However, the mechanism of icariin against ovarian cancer is poorly elucidated.

**Methods:**

Cell viability was detected to evaluate the effect of icariin on SKOV-3 cells. The cell cycle and apoptosis were analyzed. The transcript of SKOV-3 cells was profiled by RNA-seq. GSEA and DEGs analyses were performed to interpret gene expression data. Western blot and TOP/FOP flash assay were applied to detect Wnt/β-catenin signaling. MiRDB database and dual-luciferase reporter assay was applied to study the regulation of miR-1-3p on TNKS2. Anti-tumor efficacy of icariin was evaluated by xenograft mouse model. Immunohistochemistry was performed with antibodies against Ki67.

**Results:**

Icariin significantly suppressed the proliferation of SKOV-3 cells. Furthermore, icariin stalled cell cycle and induced apoptosis by blocking TNKS2/Wnt/β-catenin pathway through upregulating the level of miR-1-3p. Finally, icariin dramatically suppressed tumor growth *in vivo*.

**Conclusions:**

In this study, we demonstrated for the first time that icariin significantly attenuated the growth of ovarian tumor in xenograft mouse model. Furthermore, we systematically revealed that icariin attenuates the tumor progression by suppressing TNKS2/Wnt/β-catenin signaling *via* upregulating the level of miR-1-3p in ovarian cancer with transcriptome analysis.

## Introduction

Ovarian cancer is the deadliest female gynecological malignancy, its incidence rate ranks the second in gynecological tumor (1). Standard of care for patients diagnosed with ovarian cancer comprise cytoreductive surgery and platinum-based chemotherapy. At recurrence, chemotherapy, anti-angiogenic agents, and poly (ADP-ribose) polymerase inhibitors are used (2–6). Many novel immunotherapies are under investigation in hopes of improving clinical outcomes, but response rates remain modest (7). While significantly advances in anti-angiogenesis and PARP inhibitors treatments, the survival rates have only modestly improved (8, 9). An urgent unmet need remains for patients. Many natural compounds were widely used in cancer treatment through a variety of mechanisms with little adverse effects (10, 11).

Icariin (ICA), a flavonoid, derived from the Epimedium and was reported to exert various biological effects, including osteogenic effect, anti-inflammation, anti-oxidation, immunomodulation, and anti-cancer effects (12). It exhibiting broad cancer-preventive and/or therapeutic properties, with studies showing anti-cancer activity for many types of cancer including breast (13), ovarian (14, 15), lung (16), and gastric (17). Icariin seems to be a potential cancer therapy with pharmacological actions, including pro-apoptosis, cell cycle arrest, and immunomodulation (12). ICA’s anti-cancer mechanisms include regulating the mTOR/PI3K/AKT pathway, JAK/STAT3 pathway, NF-κB pathway, Wnt/β-catenin pathway (13, 16, 18–23). Beyond directly targeting tumor cells, ICA can also significantly inhibited the cervical tumor growth by suppressing Wnt/β-catenin signaling to promote immunity (24).

The Wnt pathway controls cell proliferation, differentiation, apoptosis, migration, as well as cell polarity, among many other biological functions (25). Many kinds of cancer, including ovarian cancer, have been observed to have deregulated Wnt/β-catenin signaling (26–28). Several ligands and related receptors were abnormally upregulated in ovarian cancer has been reported (29–31). Besides, high frequency mutation of several pathway components have been observed in ovarian cancer, such as *CTNNB1*, *AXIN1/2*, and *APC* (32, 33). These component mutations in the Wnt/β-catenin pathway indicated a poor patient survival (34). As a result, inhibiting the Wnt/β-catenin pathway was already proposed as a promising therapy option for ovarian cancer patients. Several works have been conducted to study at the putative therapeutic benefits of agents targeting this pathway, and several are now under clinical studies (35, 36).

Furthermore, some regulators play an important role in Wnt signaling transduction and were proved to be highly expressed in tumor tissue (36–41). Tankyrases are members of the PARP family and are classified into two subtypes, TNKS1 and TNKS2, which catalyzes the partial transform of many ADP-ribose into their protein substrates and are important in cell proliferation (42). TNKS stimulates the Wnt signaling pathway as an activator by increasing AXIN ubiquitination degradation and releasing β-catenin into nucleus (43, 44). It is reported that TNKS is upregulated in ovarian tumor tissue and its upregulation is negatively correlated with patient survival (45). TNKS is a novel and a promising target for cancer treatment and several inhibitors have been investigated in clinical trials of various TNKS-associated human cancers, including ovarian cancer (46). The2X-121, a small molecular targeting the PARP, as well as the tankyrases (TNKS1 and TNKS2), showed anti-tumor activity in patients with various types of solid tumor and are generally well tolerated in Phase 1 trial (NCT01618136) (47).

The microRNAs (miRNAs) belong to a class of short, endogenously-initiated non-coding RNAs that post-transcriptionally control gene expression *via* either translational repression or mRNA degradation (48). It is becoming evident that miRNAs can serve as oncomiRs by targeting tumor suppressor mRNAs and as tumor suppressor miRNAs by targeting mRNAs that encode oncoproteins (49). Studies have shown that there are various abnormal expressions of miRNA in ovarian cancer, which play a regulatory role in the development of ovarian cancer (50). For example, the high expressions of miR-182 and miR-590-3p in ovarian tumor tissue significantly promote the cell proliferation (51, 52). In addition, There are many miRNAs such as miR-506 (53, 54), miR-211 (55), miR-542-3p (56), miR-654-5p (56), miR-15a, miR-16 (57), miR-216a (58), leT-7 (59), miR-454 (60), miR-520d-3p (61), which are abnormally low-expressed in ovarian cancer, and these miRNAs play an anti-ovarian cancer role as tumor suppressors. Therefore, functionally modulating onco-miRNA and tumor-suppressive miRNA is an effective cancer treatment strategy (62). Natural products and other small molecules have been reported to exhibit anti-tumor effect *via* regulating miRNA expression (63). ICA has been demonstrated to influence several physiological and pathological processes through modulating specific miRNAs (64–66).

This study aimed to investigate the activity of ICA against ovarian cancer *in vitro* and *in vivo*, further systematically elucidating the mechanism of effect with RNA-seq combined with experiment verification.

## Materials and methods

### Cell culture

Cell Bank of Chinese Academy of Sciences (Shanghai, China) provided SKOV-3 and 293T cells. SKOV-3 cells and HEK293T cells were cultured in McCoy’s 5A (modified) Medium (Gibco) and DMEM medium (Gibco), respectively, added with 10% FBS (Gibco). Cells were kept at 37°C with 5% CO2 in a cell incubator. ICA (B21576) was purchased from Shanghai Yuanye Bio-Technology (Shanghai, China) SKL2001 was obtained from Beyotime Biotechnology (Shanghai, China). Lithium Chloride (LiCl) was purchased from Sigma (Houston, USA). In cellular experiments, ICA was dissolved with dimethyl sulfoxide (DMSO, Sigma, USA) and stored at −20 °C. For animal experiments, ICA was solved in PBS containing 0.5% Tween 80 (v/v; Sinopharm, Shanghai, China), and 1% CMC-Na (m/v; Yuanye Biotechnology, Shanghai, China).

### CCK8-proliferation assay

SKOV-3 cells were plated in a 96-well plate (3.0 × 10^3^/well). After 24 h, treated with increasing concentrations of ICA or miR-1-3p mimics. After 72 h of incubation, cells in each well were incubated with 10 μl of CCK8 (Beyotime Biotechnology, Shanghai, China). After 2 h of reaction, the OD values were detected at 450 nm. SoftMaxPro (Molecular Devices, California, USA) was used to fit a four-parameter concentration-response curve and produce and IC_50_ values.

### Preparation of RNA-seq sample and sequencing

Trizol (Invitrogen, USA) was applied to extract total RNA from SKOV-3 cells. Hieff NGSTM MaxUp Dual-mode mRNA Library Prep Kit for Illumina^®^ and NEBNext UltraTM small RNA Sample Library Prep Kit for Illumina (NEB, USA) were used to construct RNA-seq libraries. Samples were sequenced by Illumina (Illumina, USA) after library preparation. mRNA and miRNA sequencing reads were aligned using the HISAT2 and Bowtie aligner, respectively. The reads data were mapped onto the human reference genome (hg19). mRNA and miRNA transcript quantification were performed using miRDeep2 and StringTie, respectively, furthermore, differential expression analysis was performed by DESeq2.

### GSEA and target prediction analysis

GSEA was performed to interpret gene expression data using the GSEA software (GSEA version 4.1.0). Target gene prediction was conducted using the miRDB database (http://mirdb.org) database (67, 68).

### qRT-PCR

Total RNA was prepared with the Axygen^®^ AxyPrep Multisource RNA Miniprep Kit (Corning, USA) and cDNA was transcribed with the cDNA Synthesis Kits (Bio-Rad, USA) or the MicroRNA Reverse Transcription Kit (Takara, Japan). The SYBR^®^ Green Master Mix (Bio-rad, USA) or miRcute miRNA qPCR Detection Kit was used for the qRT-PCR (TIANGEN, China). For miR-1-3p, miR-4443, and miR-516a-5p, internal reference was U6. The primers were produced by Sangon Biotechnology Inc. (Shanghai, China). The sequence of primers was as follows:

U6 (Forward : CTCGCTTCGGCAGCACA, Reverse : AACGCTTCACGAATTTGCGT), hsa-miR-1-3p (Forward : CGGGCTGGAATGTAAAGAAG, Reverse : CAGCCACAAAAGAGCACAAT), hsa-miR-4443 (Forward : CGGGCTTGGAGGCGT, Reverse : CAGCCACAAAAGAGCACAAT), hsa-miR-516a-5p (Forward : CGGGCTTCTCGAGGAAAGAAG, Reverse : CAGCCACAAAAGAGCACAAT), hsa-miR-561-5p (Forward : CGGGCATCAAGGATCTTAAA, Reverse : CAGCCACAAAAGAGCACAAT).

### Plasmid construct and transfection

The 3′UTR of TNKS2 mRNA was cloned into psi-CHECK2 plasmids (GenePharma, Shanghai, China) downstream of the firefly luciferase gene and named psi-CHECK2-TNKS2-3’UTR. miRNA mimics, inhibitor, and NC of miR-1-3p were synthesized by RiboBio (Guangzhou, China). The sequences were as follows:

has-miR-1-3p mimics: 5′-UGGAAUGUAAAGAAGUAUGUAUUCACAACCUCCUAGAAAGAGUAGA-3′, has-miR-1-3p inhibitor: 5′-AUACAUACUUCUUUACAUUCCA-3′, NC Sense: 5′-UUCUUCGAACGUGUCACGUTT-3′, NC Antisense: 5′-ACGUGACACGUUCGGAGAATT-3′. Cells plated in 6-well plate were transfected with plasmid, miRNA mimics, or miRNA inhibitor in Opti-MEM medium (Gibco) using Lipofectamine 2000 (Invitrogen).

### Western blot analysis

Each sample’s total protein was extracted from treated cell and the proteins were analyzed by SDS-PAGE (69). The membranes were blocked for 2 h in TBST with 5% Bovine Serum Albumin and immunoblotted with anti-TNKS2 (1:500, ab155545, Abcam, UK), anti-β-catenin (1:1000, #8480, CST, USA), anti-Survivin (1:1000, #2803, CST, USA), anti-Cyclin D1 (1:1000, #2922, CST, USA), anti-p84 (1:1000, ab131268, Abcam, UK), and anti-α-tubulin (1:1000, #2144, CST, USA) at 4 °C overnight. After that, the membrane was incubated overnight at 4°C with Mouse Anti-Rabbit IgG H&L (1:1000, ab46540, Abcam, UK). Protein bands are treated with enhanced chemiluminescence substrates (Thermo Fisher Scientific) after standard immunoblotting procedures, and data were analyzed by the ImageQuant LAS 4000 (GE, USA). ImageJ was used to calculate protein concentrations (version 1.53a).

### Flow cytometry

Cell cycle analysis and apoptosis assay were performed as previously described with some modifications (70). A 50 g/ml propidium iodide (BD Biosciences, USA) was applied to label cells for 30 min in binding buffer. The cell cycle distribution was detected with flow cytometry (Beckman Coulter), which was then analyzed using Modfit software.

The apoptosis was studied with flow cytometry (Beckman Coulter). SKOV-3 cells were labeled by Annexin V-FITC/PI, and then was subjected to be examined. Data were analyzed using FlowJo software.

### Dual-luciferase reporter assay

We performed this assay as previously described with some modifications (71). The empty psi-CHECK2 plasmid was used as the control. Luciferase plasmids and miR-1-3p mimic or NC were transfected into 293T cells. As a positive control (PC), the miR-1-3p inhibitor gene was put into the psi-CHECK2 plasmids. The cells were extracted and examined with the Infinite M1000 fluorescent-plate reader (Tecan, USA) after 48 h after transfection.

### Wnt/β-catenin signaling detection

TOP/FOP flash reporter assay was performed this assay as previously described with some modifications (72). The TOP/FOP-Flash plasmids (GeneChem, Shanghai, China) were co-transfected into cells following ICA treatment or transfection of miRNA mimics, miRNA inhibitor, or NC. The Promega Dual-Luciferase Reporter Assay System was applied to assess fluorescence intensity after 24 h. The TOP/FOP ratio was then detected to determine the Wnt/β-catenin pathway’s activity.

### *In vivo* study

Jinan University’s Laboratory Animal Ethics Committee approved the animal experiment. Female BALB/C nude mice aged 6 weeks were obtained from HFK Biological Science (Beijing, China). The cells were then implanted subcutaneously into the right side of the axillary with 2.5 × 10^6^ SKOV-3 cells re-suspended in 0.1 ml Matrigel. Animals were given either a vehicle control or the compounds that were being studied. Mice were subsequently given intraperitoneal injections of vehicle (PBS with 0.5% Tween 80 and 1% CMC-Na), Cisplatin (2.5 mg/kg), and ICA (20 mg/kg, 40 mg/kg, 80 mg/kg). Vehicle and ICA were administered daily, and the cisplatin group twice a week. The treatment lasted for 21 days. Bodyweight and tumor volume were measured per 2 days. Tumor volume (V) was determined using the formula V = a^2^b/2, where a and b indicated the tumor’s breadth and length, respectively.

### Immunohistochemistry

After 21 days treatment, tumor tissues were collected. Tumor tissues were subsequently fixed in paraffin, and tissue slices were treated in order with the déparaffinage, rehydration, and antigen retrieval techniques. Incubation in 3% H_2_O_2_ for 10 min at room temperature stopped endogenous peroxidase. Antibodies against Ki67 were used in immunohistochemistry (Cell Signaling Technology). Finally, the signals were detected using an HRP-conjugated secondary antibody and Solarbio’s diaminobenzidine (DAB) solution. A microscope was used to photograph the slides. The ratio of positively labeled cells was used to depict the results.

### Statistical analysis

To determine the significance of various treatment groups in relation to their paired controls, one-way ANOVA and Tukey’s multiple comparisons was adopted. The mean and standard deviation are used to represent all results (SD). The *p* values were applied to illustrate significance, with *p* < 0.05 being significant. Significance was shown by the characters *: *p*< 0.05, **: *p*< 0.01, ***: *p*< 0.001, and ****: *p*< 0.0001. Significance was calculated with the of the linear trend using one-way ANOVA by GraphPad Prism (Version 9.0.0) and a test for linear trend between mean and column number.

## Results

### ICA suppress cellular proliferation of ovarian cancer SKOV-3 cells

**Figure 1:**
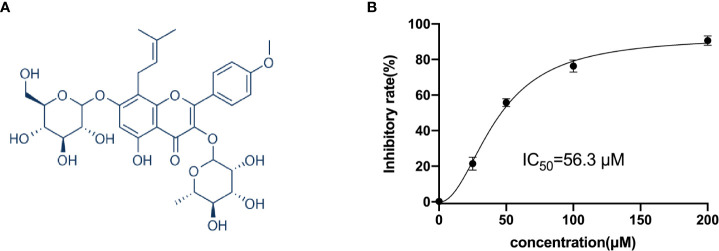
Icariin suppressed the proliferation of SKOV-3 cells. **(A)** The molecular structure of icariin. **(B)** The cells grew overnight in a 96-well plate, icariin at indicated concentrations (25, 50, 100, and 200 μM) was used to treat SKOV-3 cells for 72 h. Cell viability was assessed with the CCK8 assay. The calculated IC_50_ value was 56.3 µM. All graphs were generated with GraphPad Prism v.9.0.0. Data were shown as mean ± SD (n = 3).

ICA is a natural product that is classed as a prenylated flavonol glycoside, which is a kind of flavonoid, and its chemical structure is presented in **Figure 1A**. The cytotoxicity of ICA on SKOV-3 cells was tested to analyze the impact of ICA on ovarian cancer *in vitro*. As shown in **Figure 1B**, ICA significantly reduced SKOV-3 cellular proliferation in a concentration-dependent manner. IC_50_ value was 56.3 µM.

### ICA upregulates the expression of miR-1-3p in SKOV-3 cells

**Figure 2:**
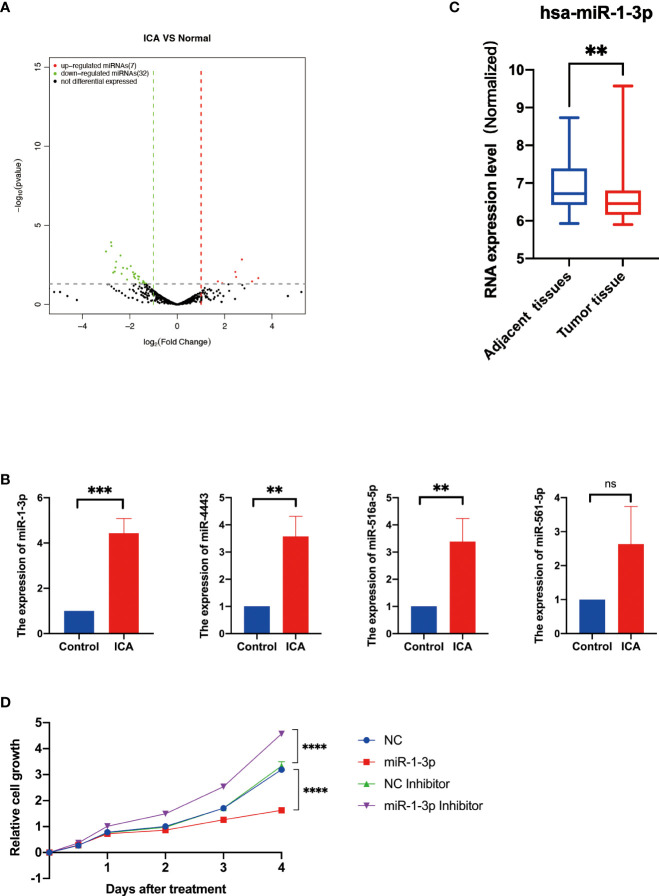
Icariin upregulates the expression of miR-1-3p in SKOV-3 cells. **(A)** The volcano plot of miRNAs with differential expression. The DESeq2 program was used to evaluate changes in gene expression using a t test. Green (downregulated) and red (upregulated) dots indicate the 39 significant differentially expressed miRNAs. **(B)** RT–qPCR analysis of miR-516-5p, miR-4443, miR-516a-5p, and miR-1-3p expression in the SKOV-3 cells after icariin treatment. (n = 3 per group). **(C)** Expression analysis of miR-1-3p in adjacent normal tissues and ovarian cancer tissues from Gene Expression Omnibus Database (GSE31801, n=121), **p <0.01. **(D)** Growth curves of SKOV-3 cells transfected with indicated miRNAs. Data were shown with mean ± SD (n = 3), ns P > 0.05, **p < 0.01, ***p < 0.001, ****p < 0.0001.

For elucidating the global impact of ICA induced alterations in gene expression patterns, we performed mRNA and miRNA sequencing for SKOV-3 cells incubated with 50 µM ICA. Control and ICA-treated samples had significantly different expressions of miRNAs (**Figure 2A**). A 32 DE miRNAs were discovered to be significantly downregulated after ICA treatment, whereas only seven miRNAs were observed to be significantly upregulated (**Supplemental Table 1**). Next, the miRNA-seq result was validated by qRT-PCR analysis. In contrast with the control, expressions of miR-516a-5p, miR-4443, and miR-1-3p in ICA treated SKOV-3 cells were remarkably increased (**Figure 2B**). The miR-1-3p, one of the elevated miRNAs, has been shown to have an anti-cancer properties in a range of malignancies, including lung, prostatic, bladder, and liver cancer. The miR-1-3p works as a tumor suppressor, which modulates the cell cycle, its overexpression can inhibit proliferation of tumor cells (73–75). We analyzed miRNA expression in existing databases (GSE31801) and found that miR-1-3p was significantly lower expressed in ovarian cancer tissues than in normal tissues (**Figure 2C**).

The viability of SKOV-3 cells was assessed to study the influence of miR-1-3p on ovarian cancer cells. As a result, miR-1-3p mimic significantly reduced SKOV-3 cell proliferation compared to NC, whereas miR-1-3p inhibitor significantly increased SKOV-3 cell growth, demonstrating that miR-1-3p overexpression can suppress SKOV-3 cell proliferation (**Figure 2D**). These results showed that ICA inhibited the proliferation of SKOV-3 cells through upregulating miR-1-3p.

### ICA suppress the WNT/β-catenin signaling through down-regulating miR-1-3p in SKOV-3 cells

**Figure 3:**
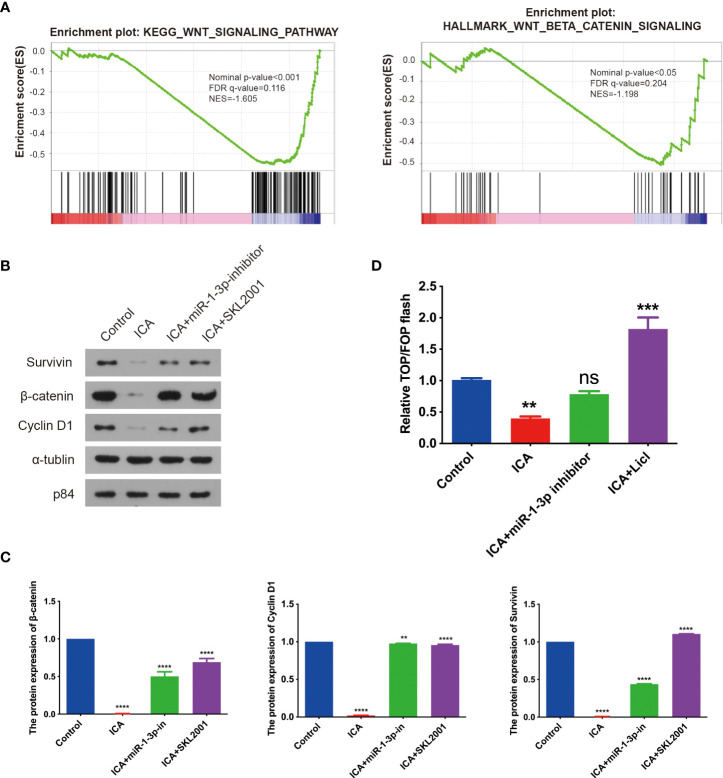
Icariin suppress Wnt/β-catenin signaling through upregulating miR-1-3p. **(A)** GSEA for the expressions of miRNAs in SKOV-3 cells treated with 50 μM icariin, revealing the action of icariin for the downregulated Wnt/β-catenin signaling pathway gene signatures. **(B, C)** The cells grew overnight in a 6-well plate, SKOV-3 cells were incubated with or without icariin (50 μM) combined with miR-1-3p inhibitor transfection or SKL2001 (50 μM) for 48 h. The expression of β-catenin, Cyclin D1, and Survivin were analyzed with western blotting. The diagram represents three separate experiments. Three independent western blotting assays were statistically analyzed. **(D)** TOP/FOP flash reporter assay in 293T cells treated with 50 μM icariin combined with miR-1-3p inhibitor transfection or LiCI for 48 h before luciferase measurement. Statistical analysis of the result from three independent assays. Data were shown with the mean ± SD, ns P > 0.05, **p < 0.01, ***p < 0.001, ****p < 0.0001.

To further elucidate mechanism of ICA against ovarian cancer, GSEA analysis was performed with global gene expression pattern in control samples and ICA-treated samples. GSEA analysis revealed that genes involved in Wnt/β-catenin pathway was markedly down-regulated in most ICA-treated cells (**Figure 3A**). Next, the effect of ICA on Wnt/β-catenin signaling were investigated. The result showed that ICA could also decrease the expression of intranuclear β-catenin, correspondingly, the expressions of cyclinD1 and Survivin were down-regulated. The inhibition of ICA on the Wnt/β-catenin pathway can be reversed by using a miR-1-3p inhibitor (**Figures 3B, C**). Top/Fop flash assay was used to further validate above result. Lithium chloride (LiCl) has formerly been shown to activate Wnt/β-catenin signaling by reducing glycogen GSK-3 and β-catenin ubiquitination (76). SKOV-3 cells were transfected with a luciferase reporter plasmid (Top flash or Fop flash), and results showed that ICA inhibited Top/Fop flash activity, whereas miR-1-3p inhibitor and Wnt/β-catenin pathway agonist SKL2001 significantly increased Top/Fop flash activity. These data indicate that ICA could inhibit Wnt/β-catenin pathway through increase the expression of miR-1-3p in SKOV-3 cells (**Figure 3D**).

### *TNKS2* is the target of miR-1-3p as an activator of Wnt/β-catenin signaling

**Figure 4:**
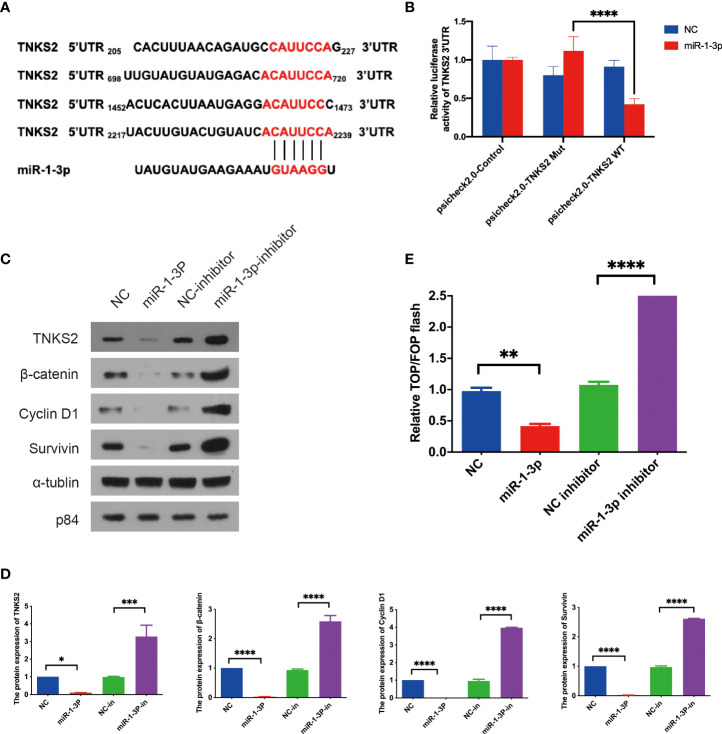
TNKS2 is the target of miR-1-3p as an activator of Wnt/β-catenin signaling. **(A)** The binding sites of miR-1-3p in 3′ UTRs of TNKS2. **(B)** Luciferase activity of TNKS2 in 293T cells was negatively regulated by the miR-1-3p. Statistical analysis of the assay of three independent assays. **(C, D)** SKOV-3 cells were transfected with indicated miRNAs. The expression of β-catenin, Cyclin D1, and Survivin were detected with western blotting after 48-h treatment. Images are representative from three independent experiments. Statistical analysis of the western blotting result from three independent assay. **(E)** A 293T cells were transfected with the indicated miRNAs combined with TOP/FOP reporter plasmids for 36 h before luciferase measurement. Cells transfected with the indicated miRNAs. Statistical analysis of the result from three independent assays, *p < 0.05, **p < 0.01, ***p < 0.001, ****p < 0.0001.

To further understand the downstream pathway of miR-1-3p, bioinformatics database (miRDB) was applied for predicting the downstream targets of miR-1-3p. Top 15 predictive target was chosen for further study (**Supplemental Table S2**). Studies have shown that the combined knockdown of the PARPs tankyrase1 (TNKS1) and TNKS2 increased the abundance of Axin1 and Axin2, as well as increasing β-catenin phosphorylation, decreasing β-catenin abundance, and then blocking Wnt/β-catenin pathway (77). In this thesis, TNKS2 was reported as an activator of Wnt/β-catenin pathway, and it was adopted for future investigation. As seen in **Figure 4A**, miR-1-3p was discovered to bind with four locations of 3′ UTR of TNKS2 according to database. To verify the regulation of miR-1-3p on TNKS2, dual-luciferase reporter assay was conducted, which indicated that miR-1-3p decreased TNKS2 luciferase activity in SKOV-3 cells (**Figure 4B**). Western blotting indicated that the expression of TNKS2 and Wnt/β-catenin pathway components were significantly attenuated by miR-1-3p mimic. Conversely, miR-1-3p inhibitor transfection markedly increased the expression of TNKS2 and Wnt/β-catenin pathway components (**Figures 4C, D**). Top/Fop flash assay confirmed that Wnt/β-catenin signaling was significantly inhibited by miR-1-3p mimic. However, the activity of pathway increased by miR-1-3p inhibitor (**Figure 4E**). In short, these findings shown that ICA elevating miR-1-3p could decrease TNKS2 expression in SKOV-3 cells, thus inactivating Wnt/β-catenin signaling.

### ICA induces cell cycle arrest and apoptosis in SKOV-3 cells *via* miR-1-3p/TNKS2/Wnt/β-catenin axis

**Figure 5:**
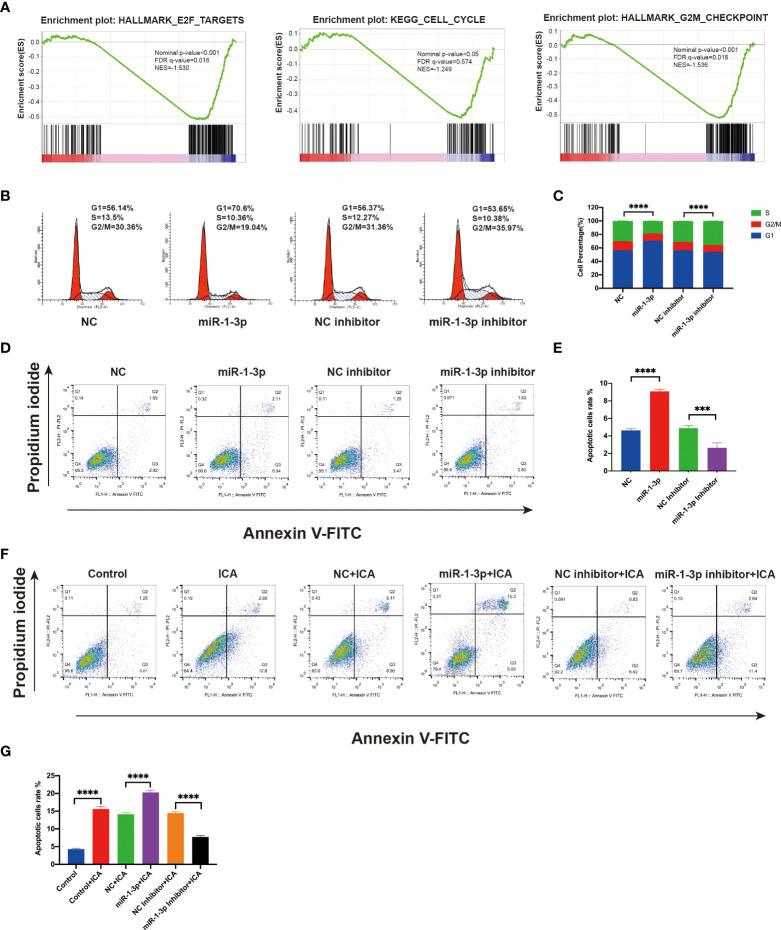
Icariin induces G1/S arrest and apoptosis in ovarian cancer cells by miR-1-3p/TNKS2/Wnt/β-catenin axis. **(A)** GSEA plots showing downregulation of the genes associated with the cell cycle. **(B)** After transfecting SKOV-3 cells with mimic or inhibitor of miR-1-3p for 48 h, flow cytometry was applied to detect cell-cycle distribution. **(C)** Statistical analysis of the result from three independent assays, ***p < 0.001. **(D)** SKOV-3 cells were transfected with the mimic or inhibitor of miR-1-3p, and after 48 h, apoptotic cells ratio was assessed using flow cytometry after Annexin V-FITC/PI labeling. **(E)** Statistical analysis of the result from three independent assays, ***p < 0.005, ****p < 0.0001. **(F)** Apoptotic cells ratio was evaluated using flow cytometry following labeling with Annexin V-FITC/PI in SKOV-3 cells treated with 50 M icariin alone or in combination with miR-1-3p mimic or miR-1-3p inhibitor transfection for 48 h. **(G)** Statistical analysis of the result from three independent assays, ***p < 0.001, ****p < 0.0001.

It is reported that Wnt/β-catenin pathway promotes cell proliferation *via* modulating the cell cycle and apoptosis (25). Previous data have shown that anti-apoptotic protein Survivin and cell cycle regulator cyclin D1 were markedly decreased in SKOV-3 cells treated with ICA or miR-1-3p mimics (**Figures 3B**, **4C**). GSEA analysis revealed that ICA significantly decreases the expression of genes associated with E2F target, cell cycle, and G2M checkpoint, which indicate the cell-cycle modulation effect of ICA (**Figure 5A**). Thus, to investigate the mechanism of ICA-induced cytotoxic effect, the effects of ICA or miR-1-3p on apoptosis and cell cycle were detected with flow cytometry. The data confirmed that miR-1-3p increased the ratio of cells in the G1/S phase, while decreased the percentage of cells in the G2/M phase. However, treatment with miR-1-3p inhibitor decreased G1/S cell proportions, while increasing G2/M cell proportions (**Figures 5B, C**). The above result revealed that miR-1-3p overexpression induced the cell cycle arrest during G1/S phase, inhibiting cell growth.

Apoptosis assay shown that the ICA or miR-1-3p markedly enhanced the amounts of apoptotic cells, while miR-1-3p inhibitor decreased the apoptotic rate of SKOV-3 cells. Besides, the ICA-induced apoptosis effect was significantly rescued by miR-1-3p inhibitor, which indicating ICA could induce apoptosis by upregulating miR-1-3p (**Figures 5D–G**). The above results confirmed that ICA induces cell cycle arrest and apoptosis in ovarian cancer cells by suppressing TNKS2/Wnt/β-catenin signaling *via* upregulating miR-1-3p.

### ICA suppresses the tumor growth in xenograft mouse model

**Figure 6:**
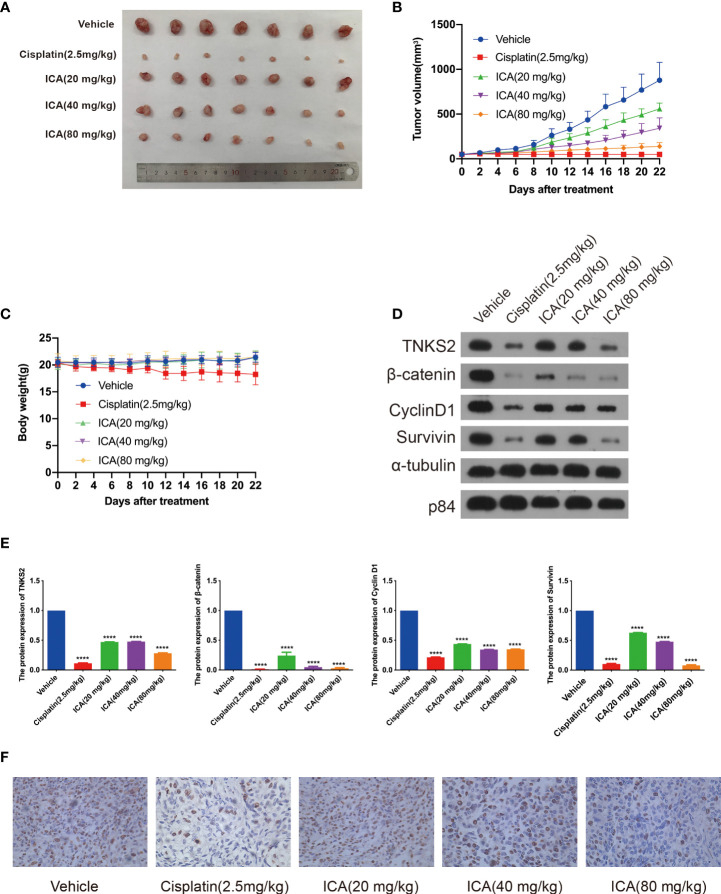
Icariin suppresses tumor growth in xenograft mouse model. **(A)** Representative photograph of the dissected tumors. **(B, C)** Tumor volume and bodyweight were detected twice a week. Data were presented as the mean ± SEM. **(D, E)** Tumors were lysed, and the indicated proteins were analyzed by Western blotting (n = 3). Data were presented as the mean ± SEM. Significance by Dunnett’s test: ****p < 0.0001. **(F)** Representative IHC images of Ki-67 expression in tumor tissue samples from the five groups, ×400 magnification.

To validate the anti-cancer effect, the *in vivo* efficacy of ICA against tumor growth was further studied. SKOV-3 cells were implanted into nude mice to establish xenograft tumor models, and animals were given different dosages of ICA (i.p., 20, 40, and 80 mg/kg) daily for 21 days. The tumors were harvested after treatment, ICA significantly reduced tumor growth compared with the vehicle group (**Figures 6A, B**), but did not affect the body weight (**Figure 6C**). Furthermore, western blotting was conducted to study the mechanism of anti-tumor effect. As is indicated in **Figures 6D, E**, ICA treatment dramatically reduced the expression of TNKS2, β-catenin, cyclinD1, and Survivin compared with the vehicle group. These findings are in-line with those of *in vitro* studies. Furthermore, cell proliferation was detected using immunocytochemistry with Ki-67 antibody, which demonstrated that the proportion of Ki-67-positive cells decreased significantly within ICA-treated group (**Figure 6F**). Those data indicated that ICA suppress the growth of tumor in ovarian cancer SKOV-3 xenograft mouse model and markedly attenuate TNKS2/Wnt/β-catenin signaling.

## Discussion

Ovarian cancer is the most dangerous cancer in gynecological tumors with highest mortality rate (1). While the introduction of targeted therapy and immunotherapy for its management, platinum-based chemotherapy remains the backbone of treatment (78). Less than 49% of affected women will live for 5 years after their diagnosis (1).

ICA possesses a variety of therapeutic potentials in several diseases, including anti-cancer capacity. ICA has been found to have broad anti-cancer characteristics in a variety of cancers through pro-apoptosis, cell-cycle regulation, anti-metastasis, anti-angiogenesis, and immunomodulation in several studies (12). Our study found that ICA exhibits significant cytotoxicity against SKOV-3 cells. Further study demonstrates that ICA significantly blockaded the TNKS2/Wnt/β-catenin signaling by upregulated miR-1-3p, accordingly, stalled cell cycle and induced apoptosis in SKOV-3 cells. Properties of ICA against ovarian cancer have been reported. According to Li et al., ICA promoted apoptosis in A2780 cells by microRNA-21 *via* activating PTEN (65). It is reported that ICA could attenuate the proliferation of SKOV-3 cells through eliciting cell cycle arrest *via* reducing FBP1/β-catenin expression (15). Those inconsistency may be attributable to the different concentrations of ICA used in their studies. In our study, the result suggested that ICA suppressed the cell proliferation in a dose-dependent manner with IC_50_ of 56.3 µM. Thus, subsequent mechanism research was carried out using 50 µM of ICA. Despite several reports about the anti-cancer effect of ICA, the impact of ICA on SKOV-3 xenograft mice model remains unknown. Furthermore, the mechanisms underlying ICA’s anti-tumor effect on ovarian cancer are yet unclear.

To systematically interpretate the mechanism of ICA against ovarian cancer, the signature of transcript in SKOV-3 cells treated with ICA were profiled through RNA-seq. We discovered that ICA significantly suppressed the expression of genes associated with Wnt/β-catenin pathway and cell cycle. Hyperactivation of Wnt/β-catenin signaling has been found in many types of cancer, particularly colorectal cancer. Critical components in Wnt signaling pathway are regarded as promising therapeutic targets, and many antagonists targeting this pathway have entered the clinical stage (79).

Wnt/β-catenin pathway is abnormally hyperactive, and downstream β-catenin and GSK3 expressions are significantly upregulated in ovarian cancer (80). Mutations in CTNNB1 (β-catenin) are identified in 16%–54% of endometrioid ovarian cancer patients, and other mutations in APC, AXIN1, and AXIN2, which are key downstream proteins of the Wnt/β-catenin pathway, are also found in different types of ovarian cancer (32, 79). Based on the deregulation of this pathway in ovarian cancer, targeting the Wnt signaling pathway is believed to be a potential ovarian cancer therapy approach. Clinical trials have showed that combining the Wnt/β-catenin pathway inhibitor WNT974 with cisplatin can effectively induce cytotoxic effects and destroy ovarian cancer cells, demonstrating good anti-tumor efficacy (81). RSPO protein binds to Wnt protein to activate β-catenin signaling, and anti-RSPO monoclonal antibody can significantly inhibit tumor growth in ovarian cancer mouse PDX model (82). Niclosamide, an FDA-approved anthelmintic, suppresses ovarian cancer stem cell proliferation by blocking the Wnt/β-catenin signaling *via* DVL2 and LRP6 inhibition (83). Furthermore, several bioactive chemicals originated from natural products have anti-cancer properties by blocking the Wnt/β-catenin signaling pathway (84). Studies have indicated that inhibiting the TLR4/MyD88/NF-B and Wnt/β-catenin pathways inhibits the growth of cervical tumor (24).. In our study, we discovered that ICA inhibited the proliferation of ovarian cancer cells through blocking Wnt/β-catenin signaling.

Here, miRNA-seq analysis suggested that in SKOV-3 cells treated with ICA, seven miRNAs were markedly upregulated, and 32 miRNAs were significantly down-regulated. Literature investigation and analysis of significantly differentially expressed miRNAs in reports found that significantly upregulated miR-1-3p, miR-516a-5p, miR-561-5p, and miR-4443 have been shown to have the function of tumor suppressor in a variety of studies. In ICA-treated SKOV-3 cells, qPCR verification suggested that miR-1-3p, miR-516a-5p, and miR-4443 markedly increased. Target genes were predicted by the above three miRNAs in the miRDB miRNA database, and the top 15 Target genes with high scores were selected for follow-up analysis according to Target Score sequencing of each miRNA. GSEA analysis revealed that ICA significantly reduced the expression of genes associated with Wnt/β-catenin pathway, indicating that ICA may block Wnt/β-catenin signaling, causing apoptosis and cell cycle arrest. End-anchor polymerase TNKS2, which is regulated by miR-1-3p, has been reported to enhance cytoplasmic β-catenin aggregation, therefore controlling the Wnt/β-catenin signaling (25, 41, 79). It was reported that TNKS2 promotes Wnt/β-catenin signaling in ovarian cancer, increasing tumor cell glycolysis and proliferation (45).

The miR-1-3p belongs to miR-1 family member, which has been shown to be a tumor suppressor in a range of cancers, including lung cancer and colon cancer (85). miR-1-3p can limit the proliferation of J82 cells *via* the upregulation of SFRP1, which is down-regulated in bladder tumor tissue (73). It has been found that down-regulation of miR-1-3p indicates a poor prognosis in patients, and miR-1-3p overexpression in 22RV1 and LncaP cells has been proven to induce cell-cycle arrest and limits tumor cell growth. The miR-1-3p has been shown in studies to bind the 3’UTR of cycle-regulating genes E2F5 and PFTK1 and to decrease their mRNA and protein expression (74). The miR-1-3p is significantly low expressed in lung adenocarcinoma tumor tissues, and overexpression of miR-1-3p in lung cancer cells can suppress tumor growth, invasion, and migration. Further studies have found that PRC is an miR-1-3p target, and inhibiting PRC1 expression may have an anti-lung cancer effect (86). The miR-1-3p is considerably downregulated in colon tumor tissues, and its overexpression can dramatically decrease colon cancer cell proliferation and invasion by downregulating STC2 (87). Another study on colon cancer found that LINC00242 and G6PD were significantly overexpressed in colon cancer. Functional studies found that LINC00242 enhanced the glycolysis pathway of tumor cells and promoted cell proliferation through increasing the expression of G6PD *via* reducing the expression of miR-1-3p. Increased miR-1-3p can suppress G6PD, promote apoptosis, reduce tumor cell glycolysis, and inhibit cell proliferation (88). The miR-1-3p was chosen for further investigation since it was discovered to be irregularly upregulated in numerous types of malignancies and to have a tumor suppressor role in the previous study. To clarify the function of miRNA in SKOV-3 cells, we investigated the effect of miR-1-3p on proliferation, apoptosis, and cycle distribution and whether it mediates the anti-tumor effect of ICA. The findings demonstrate that miR-1-3p significantly suppressed SKOV-3 cell proliferation, induced apoptosis, and induced G1/S phase arrest, and that miR-1-3p inhibitor reversed ICA’s apoptosis-inducing effect, implying that ICA plays an anti-ovarian cancer role by upregulating tumor suppressor miR-1-3p. Yet, the mechanism by which ICA regulates miR-1-3p remains unknown.

We demonstrated that miR-1-3p may bind to the 3’UTRs of TNKS2 and decrease its expression. The small-molecule XAV939 has been demonstrated in studies to inhibit TNKS1 and TNKS2, to stabilize AXIN, and to accelerate β-catenin degradation, which lead to inhibition of transcriptional regulation (77). Subsequent studies have found that TNKS inhibitors such as IWR-1, IWR-2, and JW55 can stabilize AXIN and inhibit the proliferation inhibition mediated by Wnt/β-catenin signaling on many types of tumor cells, including colorectal and liver cancer. In ovarian tumor tissues, TNKS2 has been discovered to be significantly overexpressed (45), and PARP, including TNKS1 and TNKS2, are important targets for tumor therapy. Currently, a variety of PARP inhibitors have been entered into clinical studies or marketed for ovarian cancer treatment (46).

Given the crucial role of Wnt signaling in immune system, inhibiting this pathway can improve anti-tumor immunity in ovarian cancer (89). ICA boosted anti-tumor immunity through a variety of mechanisms, including modulation of Wnt pathway (24, 90). In our study, *in vivo* assay indicated that ICA significantly suppressed tumor growth in xenograft mouse model. The anti-tumor efficacy of ICA may be due to the synergistic effect of targeting cancer cells and enhancement of immunity.

## Conclusions

In conclusion, our study demonstrated that ICA inhibits the growth of ovarian cancer SKOV-3 cells *in vitro* and *in vivo*. Further investigation systematically revealed that ICA induces cell-cycle arrest and apoptosis in SKOV-3 cells by blocking TNKS2/Wnt/β-catenin signaling *via* the tumor-suppressor miR-1-3p with transcriptome analysis. This study provides a clue to fully reveal the anti-ovarian cancer effect of ICA.

## Data availability statement

The data presented in the study are deposited in the https://www.ncbi.nlm.nih.gov/sra repository, accession number PRJNA843462.

## Ethics statement

The animal study was reviewed and approved by Laboratory Animal Ethics Committee of Jinan University.

## Author contributions

YF, HL, and JQ participated in the research design. YF and HL conducted the experiments. YF, HL, and ML performed the data acquisition and analysis. LS, ZM, SL, and YZ contributed to the investigation and statistical analysis. YF and JQ wrote or contributed to the writing of the manuscript. All authors contributed to the article and approved the submitted version.

## Funding

This work was supported by the National Natural Science Foundation of China (NO: 82174147) and the Science and Technology Planning Project of Guangdong Province (NO: 2017A020213001).

## Conflict of interest

The authors declare that the research was conducted in the absence of any commercial or financial relationships that could be construed as a potential conflict of interest.

## Publisher’s note

All claims expressed in this article are solely those of the authors and do not necessarily represent those of their affiliated organizations, or those of the publisher, the editors and the reviewers. Any product that may be evaluated in this article, or claim that may be made by its manufacturer, is not guaranteed or endorsed by the publisher.
