# Supplementary material for: Icariin attenuates the tumor growth by targeting miR-1-3p/TNKS2/Wnt/β-catenin signaling axis in ovarian cancer
Source: Front Oncol. 2022 Sep 14;12:940926. doi: 10.3389/fonc.2022.940926 (PMC9516086; doi:10.3389/fonc.2022.940926)
Supplement: Supplementary file 1 [file Table_1.docx]

Table S1. Significantly differentially expressed miRNA after icariin treatment

| miRNA | MeanCPM  (ICA) | MeanCPM  (Normal) | log2(ICA/Normal) | log2FoldChange | pvalue | qvalue | result |
| --- | --- | --- | --- | --- | --- | --- | --- |
| hsa-let-7a-3p | 135.08 | 336.78 | -1.3180 | -1.4375 | 0.0321 | 1 | down |
| hsa-let-7f-1-3p | 4.81 | 19.35 | -2.0082 | -2.1210 | 0.0054 | 0.67681 | down |
| hsa-miR-1-3p | 10.19 | 1.4 | 2.8637 | 2.7260 | 0.0014 | 0.33008 | up |
| hsa-miR-1303 | 4.25 | 13.49 | -1.6664 | -1.7814 | 0.0211 | 1 | down |
| hsa-miR-135b-5p | 24.92 | 60.2 | -1.2725 | -1.3914 | 0.0437 | 1 | down |
| hsa-miR-155-5p | 23.5 | 58.29 | -1.3106 | -1.4292 | 0.0387 | 1 | down |
| hsa-miR-15b-3p | 74.19 | 183.41 | -1.3058 | -1.4251 | 0.0344 | 1 | down |
| hsa-miR-196b-5p | 7.36 | 24.18 | -1.7160 | -1.8321 | 0.0125 | 0.96123 | down |
| hsa-miR-20a-5p | 473.75 | 1706.16 | -1.8486 | -1.9681 | 0.0037 | 0.63592 | down |
| hsa-miR-20b-5p | 4.81 | 15.78 | -1.7140 | -1.8277 | 0.0173 | 1 | down |
| hsa-miR-26b-5p | 768.26 | 1769.93 | -1.2040 | -1.3236 | 0.0465 | 1 | down |
| hsa-miR-32-5p | 4.53 | 12.6 | -1.4758 | -1.5904 | 0.0431 | 1 | down |
| hsa-miR-335-3p | 0.85 | 5.22 | -2.6185 | -2.7055 | 0.0094 | 0.87049 | down |
| hsa-miR-3619-5p | 1.00E-04 | 1.53 | -13.9012 | -6.1787 | 0.0141 | 1 | down |
| hsa-miR-374a-5p | 78.72 | 221.97 | -1.4956 | -1.6149 | 0.0170 | 1 | down |
| hsa-miR-374a-3p | 24.64 | 71.53 | -1.5375 | -1.6565 | 0.0163 | 1 | down |
| hsa-miR-4443 | 3.68 | 0.89 | 2.0478 | 1.9020 | 0.0457 | 1 | up |
| hsa-miR-451a | 1.13 | 6.62 | -2.5505 | -2.6417 | 0.0082 | 0.87049 | down |
| hsa-miR-454-3p | 11.61 | 39.07 | -1.7507 | -1.8684 | 0.0094 | 0.87049 | down |
| hsa-miR-4728-3p | 1.42 | 5.73 | -2.0126 | -2.1178 | 0.0270 | 1 | down |
| hsa-miR-516a-5p | 5.38 | 0.89 | 2.5957 | 2.4466 | 0.0085 | 0.87049 | up |
| hsa-miR-551b-5p | 1.42 | 6.75 | -2.2490 | -2.3530 | 0.0115 | 0.93649 | down |
| hsa-miR-561-5p | 10.48 | 2.93 | 1.8387 | 1.7127 | 0.0343 | 1 | up |
| hsa-miR-577 | 10.48 | 26.47 | -1.3367 | -1.4549 | 0.0436 | 1 | down |
| hsa-miR-590-3p | 1.98 | 14.76 | -2.8981 | -3.0018 | 0.0004 | 0.15214 | down |
| hsa-miR-610 | 1.00E-04 | 1.27 | -13.6325 | -5.9197 | 0.0348 | 1 | down |
| hsa-miR-6720-5p | 1.00E-04 | 1.27 | -13.6325 | -5.9197 | 0.0348 | 1 | down |
| hsa-miR-7974 | 14.73 | 46.46 | -1.6572 | -1.7756 | 0.0115 | 0.93649 | down |
| hsa-miR-888-5p | 11.89 | 76.24 | -2.6808 | -2.7976 | 0.0001 | 0.08109 | down |
| hsa-miR-890 | 7.65 | 48.24 | -2.6567 | -2.7734 | 0.0002 | 0.09127 | down |
| hsa-miR-891a-5p | 15.01 | 72.8 | -2.2780 | -2.3961 | 0.0008 | 0.21807 | down |
| hsa-miR-891b | 1.42 | 8.02 | -2.4977 | -2.6016 | 0.0046 | 0.67681 | down |
| hsa-miR-892a | 2.55 | 14.13 | -2.4702 | -2.5795 | 0.0020 | 0.39615 | down |
| hsa-miR-892b | 2.83 | 12.86 | -2.1840 | -2.2928 | 0.0049 | 0.67681 | down |
| hsa-miR-95-3p | 7.93 | 22.91 | -1.5306 | -1.6476 | 0.0253 | 1 | down |
| hsa-novel-15-mature | 3.96 | 0.64 | 2.6294 | 2.4803 | 0.0183 | 1 | up |
| hsa-novel-30-star | 1.42 | 0.13 | 3.4493 | 3.1555 | 0.0348 | 1 | up |
| hsa-novel-60-mature | 1.7 | 0.13 | 3.7090 | 3.4146 | 0.0219 | 1 | up |
| hsa-novel-86-mature | 0.28 | 12.47 | -5.4769 | -5.4694 | 0.0000 | 0.00212 | down |
