# Supplementary material for: Icariin attenuates the tumor growth by targeting miR-1-3p/TNKS2/Wnt/β-catenin signaling axis in ovarian cancer
Source: Front Oncol. 2022 Sep 14;12:940926. doi: 10.3389/fonc.2022.940926 (PMC9516086; doi:10.3389/fonc.2022.940926)
Supplement: Supplementary file 2 [file Table_2.docx]

Table S2. Predictive targets of miR-1-3p

| Target Rank | Target Score | miRNA Name | Gene Symbol | Gene Description |
| --- | --- | --- | --- | --- |
| 1 | 100 | hsa-miR-1-3p | [HACD3](http://www.ncbi.nlm.nih.gov/entrez/query.fcgi?db=gene&cmd=Retrieve&dopt=full_report&list_uids=51495) | 3-hydroxyacyl-CoA dehydratase 3 |
| 2 | 100 | hsa-miR-1-3p | [MMD](http://www.ncbi.nlm.nih.gov/entrez/query.fcgi?db=gene&cmd=Retrieve&dopt=full_report&list_uids=23531) | monocyte to macrophage differentiation associated |
| 3 | 100 | hsa-miR-1-3p | [SLC44A1](http://www.ncbi.nlm.nih.gov/entrez/query.fcgi?db=gene&cmd=Retrieve&dopt=full_report&list_uids=23446) | solute carrier family 44 member 1 |
| 4 | 99 | hsa-miR-1-3p | [PAX7](http://www.ncbi.nlm.nih.gov/entrez/query.fcgi?db=gene&cmd=Retrieve&dopt=full_report&list_uids=5081) | paired box 7 |
| 5 | 99 | hsa-miR-1-3p | [CDK14](http://www.ncbi.nlm.nih.gov/entrez/query.fcgi?db=gene&cmd=Retrieve&dopt=full_report&list_uids=5218) | cyclin dependent kinase 14 |
| 6 | 99 | hsa-miR-1-3p | [CPED1](http://www.ncbi.nlm.nih.gov/entrez/query.fcgi?db=gene&cmd=Retrieve&dopt=full_report&list_uids=79974) | cadherin like and PC-esterase domain containing 1 |
| 7 | 99 | hsa-miR-1-3p | [SMIM14](http://www.ncbi.nlm.nih.gov/entrez/query.fcgi?db=gene&cmd=Retrieve&dopt=full_report&list_uids=201895) | small integral membrane protein 14 |
| 8 | 99 | hsa-miR-1-3p | [GJA1](http://www.ncbi.nlm.nih.gov/entrez/query.fcgi?db=gene&cmd=Retrieve&dopt=full_report&list_uids=2697) | gap junction protein alpha 1 |
| 9 | 99 | hsa-miR-1-3p | [TAGLN2](http://www.ncbi.nlm.nih.gov/entrez/query.fcgi?db=gene&cmd=Retrieve&dopt=full_report&list_uids=8407) | transgelin 2 |
| 10 | 99 | hsa-miR-1-3p | [TNKS2](http://www.ncbi.nlm.nih.gov/entrez/query.fcgi?db=gene&cmd=Retrieve&dopt=full_report&list_uids=80351) | tankyrase 2 |
| 11 | 99 | hsa-miR-1-3p | [XPO6](http://www.ncbi.nlm.nih.gov/entrez/query.fcgi?db=gene&cmd=Retrieve&dopt=full_report&list_uids=23214) | exportin 6 |
| 12 | 99 | hsa-miR-1-3p | [GLCCI1](http://www.ncbi.nlm.nih.gov/entrez/query.fcgi?db=gene&cmd=Retrieve&dopt=full_report&list_uids=113263) | glucocorticoid induced 1 |
| 13 | 98 | hsa-miR-1-3p | [TWF1](http://www.ncbi.nlm.nih.gov/entrez/query.fcgi?db=gene&cmd=Retrieve&dopt=full_report&list_uids=5756) | twinfilin actin binding protein 1 |
| 14 | 98 | hsa-miR-1-3p | [SS18](http://www.ncbi.nlm.nih.gov/entrez/query.fcgi?db=gene&cmd=Retrieve&dopt=full_report&list_uids=6760) | SS18, nBAF chromatin remodeling complex subunit |
| 15 | 98 | hsa-miR-1-3p | [SNX2](http://www.ncbi.nlm.nih.gov/entrez/query.fcgi?db=gene&cmd=Retrieve&dopt=full_report&list_uids=6643) | sorting nexin 2 |
